# Lady House Surgeons

**Published:** 1901-11-16

**Authors:** 


### LADY HOUSE SURGEONS.

The question of the lady doctor has reached a somewhat acute stage at Macclesfield, where the honorary medical staff of the infirmary has resigned in consequence of the Governors having appointed a lady as junior house surgeon. It is a great pity that this question, which has cropped up with increasing frequency during the last few years, and will probably do so still more often in years to come, cannot be discussed and decided on its merits without introducing the ever-aggravating problem of the eternal feminine. In appointing a house surgeon the Governors have nothing whatever to do with the rights of women or with the equality of the sexes, nor should they allow sympathy with what they may regard as the weaker side to influence them in their choice. The question is entirely one of suitability for the post, and we cannot believe that any decent men who have any knowledge of the sort of duties which house surgeons have to perform would for a moment imagine that a woman is a fit and proper person to act as house surgeon to a general hospital. There are many professional posts to which women may be properly appointed. Women doctors have made their mark, and no one would wish to raise the question of sex against them. But there are certain positions in regard to which the question of sex intrudes itself whether we will or no, and the position of house surgeon at a small general hospital, where each officer must be prepared to undertake every kind of work, is one of that nature. There are plenty of posts open to women. They may, with full propriety, become house surgeons at hospitals for women or for children, or even at institutions into which patients of both sexes are received, when the staff is sufficiently large to render it possible to arrange for a proper distribution of duties. But at a small general hospital, where often the chief reason for having more than one resident is that there may always be one on the spot to attend to any emergency that may arise, the question assumes a totally different aspect. We may as well put the matter plainly. A considerable proportion of the patients at all accident hospitals are men, often young men, who, except for the mere local trouble caused by their injury, are full of health and vigour, and we maintain without fear of contradiction that there are certain things which sometimes require to be done for such patients which women cannot do.

According to ordinary arrangements—that is, with male house surgeons and female nurses—while there is much in regard to the treatment of women which the house surgeons leave to the nurses, there is much in regard to the male patients which house surgeons of any decency either do or superintend themselves rather than throw upon the nurses. Both men and women being engaged in the treatment of the sick, wherever the tone is good the duties are so divided that decency prevails. But if a lady house surgeon is to be appointed at a small hospital where in the absence of her male colleague she must do everything that comes, then difficulties will arise, and the patients will suffer. If there is anything in sex at all, the males of Macclesfield are being badly used. A woman going to hospital knows that although the doctor may be a man she will be surrounded by nurses of her own sex.

But a man of Macclesfield, wherever he may be hurt or however delicate his malady, will know that when he applies at the Infirmary he may be thrown entirely into the hands of women; and that seems to us an impracticable position of affairs. Of course there are many social difficulties connected with the appointment of lady house surgeons, but to these we do not refer, as we think the decision should be founded on other grounds, namely, on what is best for the patients. There is one point, however, to which we would draw attention, namely, that the life of two house surgeons living together, sharing each other's interests, and helping each in their work as the best house surgeons do, is one of considerable intimacy; and although there no doubt will always be men who will be willing to accept such a position, it is extremely improbable that the best men, or for that matter the best women, will ever allow themselves to be forced into such intimate companionship with persons of the opposite sex who are not of their own choosing. There is plenty of useful medical work for medical women to do. Let them do it.

We would be the last to put any hindrance in their way or in the slightest degree to suggest any question of inferiority. In their own field they are probably equal if not superior to men. But not as house surgeons to general hospitals.

